# Attitudes towards human milk banking among native turkish and refugee women residing in a rural region of Turkey: a mixed-methods approach

**DOI:** 10.1186/s13006-022-00516-2

**Published:** 2022-10-27

**Authors:** Ceren Varer Akpinar, Aliye Mandiracioglu, Safiye Ozvurmaz, Filiz Adana, Nazife Koc, Fatma Kurt

**Affiliations:** 1 Faculty of Medicine, Department of Public Health, Giresun University, Giresun, Turkey; 2 Faculty of Medicine, Department of Public Health, Ege University, Izmir, Turkey; 3 Faculty of Nursing, Department of Public Health Nursing, Adnan Menderes University, Aydın, Turkey; 4 Home Patient Care Services, Istanbul Rumeli University, İstanbul, Turkey

**Keywords:** Human milk banking, Breastfeeding, Wet nursing, Qualitative

## Abstract

**Background:**

Human milk banks (HMB) play an essential role by providing human milk to infants who would otherwise not be able to receive mother’s milk. There is currently no donor milk bank in Turkey. For any new health intervention to be successful, determining its acceptability is a vital first step. This study intends to determine the opinions, knowledge, and attitudes of native Turkish and refugee women living in Çeştepe, Aydın, a rural area in Turkey, about HMB.

**Methods:**

A population-based cross-sectional, mixed-methods study was conducted. Qualitative study data were collected through in-depth interviews with 33 women, and quantitative study data were collected using a questionnaire. A total of 271 women in the region were included in the study. Qualitative data were thematically analyzed and then a conceptual framework was created. Logistic regression was performed for quantitative data.

**Results:**

Fifty-seven point nine per cent of the women were willing to donate breast milk, whereas only 27.7% were willing to use donor milk for their babies. Religious concerns, fear of infectious diseases, and distrust in people they did not know were among the reasons for the negative attitudes of the women. Fear of infectious diseases was mentioned specifically by native Turkish women, and religious concerns were reported both by native Turkish and refugee women. The importance of breast milk and religion were among the reasons for positive attitudes. Additionally, odds of having a positive attitude were 4.19 times higher in homemaker women (95% CI 2.0, 8.76); 4.77 times higher in women with three or more children (95% CI 1.25, 8.15); 6.12 times higher in women who had a positive attitude towards wet nursing (95% CI 3.14, 9.63); and 2.68 times higher in those who had previously heard about human milk banking (95% CI 1.24, 5.79).

**Conclusion:**

Attitudes towards HMB are affected by religion, cultural beliefs, and concerns about the safety of breast milk in HMBs. Refugees and native Turkish women are found to have similar religious concerns. These findings should be taken into consideration in human milk banking initiatives and in activities to increase acceptance by the public.

## Background

Breast milk meets all nutritional needs of a term baby in its first six months of life. Not every baby can have breast milk in desired amounts for several reasons. Due to reasons concerning the mother or the baby such as severe infections, use of medications, hepatitis B, hepatitis C, tuberculosis, substance use, premature birth, and low birth weight, breastfeeding is sometimes not possible [1, 2]. When breastfeeding is not possible, the World Health Organization (WHO) recommends the use of donated breast milk as the best feeding option [3, 4]. This brings forward the concepts of wet nursing and human milk banking (HMB). A wet nurse refers to a mother who breastfeeds someone else’s baby. Wet nursing is a method that has been used as an alternative to breastfeeding for many years. In nations where the majority of the population is Muslim, like Turkey, a wet nurse is generally a relative or a family friend who knows the baby’s family [5]. Muslims believe that when a mother breastfeeds someone else’s baby as a wet nurse, then these people become relatives (including all family and children). The baby receiving the milk and the donor mother’s children become milk siblings. This is also mentioned in the Quran, the holy book of Muslims, and milk siblings are not allowed to wed [6]. Wet nursing has been decreasing due to various reasons including the difficulty of finding wet nurses, growing concerns regarding infectious diseases, wider use of feeding bottles, wider use of infant formulas, and increased availability of animal milk [7].

The idea of HMB emerged in the 1900s as an alternative to wet nursing [8]. The goal of HMB is to collect milk from lactating mothers and to help feed other women’s babies. HMB is the process by which donor breast milk is collected, screened, processed, and stored, thus providing a source of human breast milk for infants who would otherwise not receive it [9]. In Brazil, with the integration of human milk banks into newborn health policies, newborn mortality has been reduced by almost three-quarters [10]. Although Islamic traditions accept breast milk as an ideal source of nutrition for infants, Muslim societies have reservations about human milk banks due to religious concerns [11].

According to the 2018 national data in Turkey, 40.7% of children under the age of six months are exclusively breastfed since birth. Additionally, contrary to the recommendation that children under six months should be exclusively breastfed, 23% of children receive breast milk with other milk and 12% of children receive complementary foods in addition to breast milk [12]. In 2011, the “Turkish Human Milk Banking Model” was developed according to the relevant standards in Turkey [13]. However, due to differences in opinions and the reason that it was not accepted by the public, no further work was done. A study conducted on Turkish religious clergy in 2015 demonstrated that most of them did not approve of founding Western-style milk banks [13].

Acceptance by the public is a vital first step for any new health intervention to be successful. In this regard, opinions, recommendations, and attitudes of women will guide in creating models of HMB. Several hospital-based studies conducted in Turkey reported on the knowledge and attitudes of women about HMB [14–16]. However, little is known about the knowledge and attitudes of women regarding HMB in rural areas where breastfeeding is a strong tradition. Furthermore, there are more than 3,000,000 registered refugees in Turkey and the majority of these refugees are Muslims [17]. There is no information about the refugees’ knowledge and attitudes towards HMB in Turkey. Given the different socio-cultural structures in Turkey, determining the reasons for attitudes and knowledge of both native Turkish and refugee women about HMB is important for better planning of the practices of HMB in Muslim countries.

The qualitative part of this study intends to determine the opinions on HMB of native Turkish women and refugees living in a rural area called Çeştepe in Aydın, Turkey, and whether HMB was acceptable for them. The quantitative part of this study intends to determine these women’s knowledge and attitudes toward HMB and to establish the factors associated with HMB attitudes.

## Methods

A population-based cross-sectional, mixed-methods study was conducted. A mixed-methods approach was used with qualitative and quantitative data collection and analysis. The qualitative study preceded the quantitative study and provided insight and a deeper understanding of the native Turkish and refugee women’s perceptions and attitudes about human milk and HMB. The quantitative study subsequently enabled the quantification of the issues identified through the qualitative study. The study was conducted in a rural area called Çeştepe in Aydın. Çeştepe is a fertile agricultural area in western Turkey. The distance between Çeştepe and the city center is two km. The population of Çeştepe is 5767 (2905 males, 2862 females). The main source of income is agriculture because agricultural lands are common in the area. The area receives migrants both from within the country and abroad; contains different socio-cultural structures of the country; and has refugees from a variety of ethnic groups. Most of the migrants in Çeştepe came many years ago from the Balkans (Macedonians, Yugoslavs, Bulgarians) and have the same socio-cultural structure as that of local people. The region has been hosting refugees for the last ten years, especially after the Syrian War. The refugees are mostly from Syria, but some come from Iraq and Afghanistan. According to the information obtained from the headman of that region, approximately 60 refugee families live in the region.

### Study population

Purposive sampling was used to select the participants for qualitative interviews. This sampling technique was preferred because it allowed a purposive selection of participants who were knowledgable about breastfeeding and infant feeding practices, through personal experiences. The women were reached through the auspices of the headman of the region and the researcher who was working as a midwife in a primary health care institution in the region. When most of the data started to be similar, a saturation point was assumed to have been reached and data collection was ended. In this part of the study, IDIs were conducted with 33 women. Fifteen of these women with whom IDIs were conducted were refugees, eight were Syrians, four were Iraqis, and three were Afghans.

All women who were aged over 18 years and had a birth in the last five years in Çeştepe, Aydın, were included in the quantitative study. According to the data registered with the primary healthcare institutions, there were 324 women aged over 18 years who had given birth within the last five years. Even if they were not registered in primary health institutions, the contact information of women who presented even once to hospitals was recorded. No sampling was performed and 291 women who were not included in the qualitative part of the study were invited to join the cross-sectional study. Two hundred seventy-one (93.1%) women agreed to participate in the study.

### Data collection

In the qualitative part of this study, with an interpretive approach, a case study design was used to determine the perceptions of women about HMB milk donation and use of donor breast milk; obstacles and facilitators for its acceptability; and the ways to support breast milk donation and banking in the future. Qualitative study data were collected with in-depth interviews (IDIs) performed using a semi-structured interview form with seven open-ended questions, between the dates of December 2021 and January 2022. The questions that we used in IDIs were as follows: Do you know the meaning of breast milk banking and breast milk donation? Would you donate your own milk? Would you feed your baby with donated breast milk? Reasons for your positive / negative thoughts? Do you have any information on practices related to breast milk donation and use? What can be done to make sure donated breast milk is safe? Would you like to establish a breast milk bank in our country? Each IDI began with an explanation of the purpose of the study and obtaining informed voluntary consent from the participants. Interviews were conducted by an experienced researcher (NK) and another researcher (FK) who made audio recordings and took notes. IDIs were conducted in different sessions for each woman. Each IDI lasted 45 min to 1 h. All interviews were conducted in the local language and interviews with people whose mother tongue was Arabic were performed with the help of Arabic interpreters. Opinions of women who had IDIs were shown with their respondent codes, ages, and refugee status, native (N) or refugee (R).

Cross-sectional study data were collected using a questionnaire in in-person interviews with women between February and March 2022. Data were collected in the participants’ homes. The questionnaire was developed by the researchers based on a literature search and in line with the thematic and conceptual framework determined with the qualitative method. Questions were designed to determine the knowledge and attitudes of women regarding wet nursing and HMB and their sociodemographic characteristics. The survey consisted of 27 multiple choice questions and lasted approximately 20 min. The questionnaire was prepared in the local language and interviews with people whose mother tongue was Arabic were performed with the help of Arabic interpreters.

The dependent variable of the quantitative part of the study was the attitudes of women towards HMB. Attitudes of women toward HMB were determined using two variables: acceptance of breast milk donation or acceptance of the use of donor milk to feed an infant. A woman was considered as having a positive attitude toward milk banking if she was accepting of breast milk donation for banking and / or she was accepting of the use of donor milk to feed an infant. Independent variables of the study were age, education, family type, number of children, knowledge and attitudes toward wet nursing, and knowledge about HMB. A mother was considered to be aware of HMB if she had heard about HMB. A mother was considered to be aware of wet nursing if she had heard about wet nursing. Respondents’ attitudes toward wet nursing were evaluated using four variables: previously being a wet nurse for a baby, willingness to be a wet nurse for a baby, having a wet nurse for her own baby, and willingness to have a wet nurse for her own baby.

### Ethical considerations

The study was conducted according to the Helsinki Declaration for Ethical Principles of Research. Approval was obtained from the Clinical Research Ethics Committee of Ege University Medical School (17.12.2021-463216-924). Additionally, participants were asked to sign informed voluntary consent forms and necessary information about the study was given. The qualitative part of the study was reported according to the Consolidated Criteria for Reporting Qualitative Research (COREQ) checklist [18].

### Statistical analysis

**Fig. 1:**
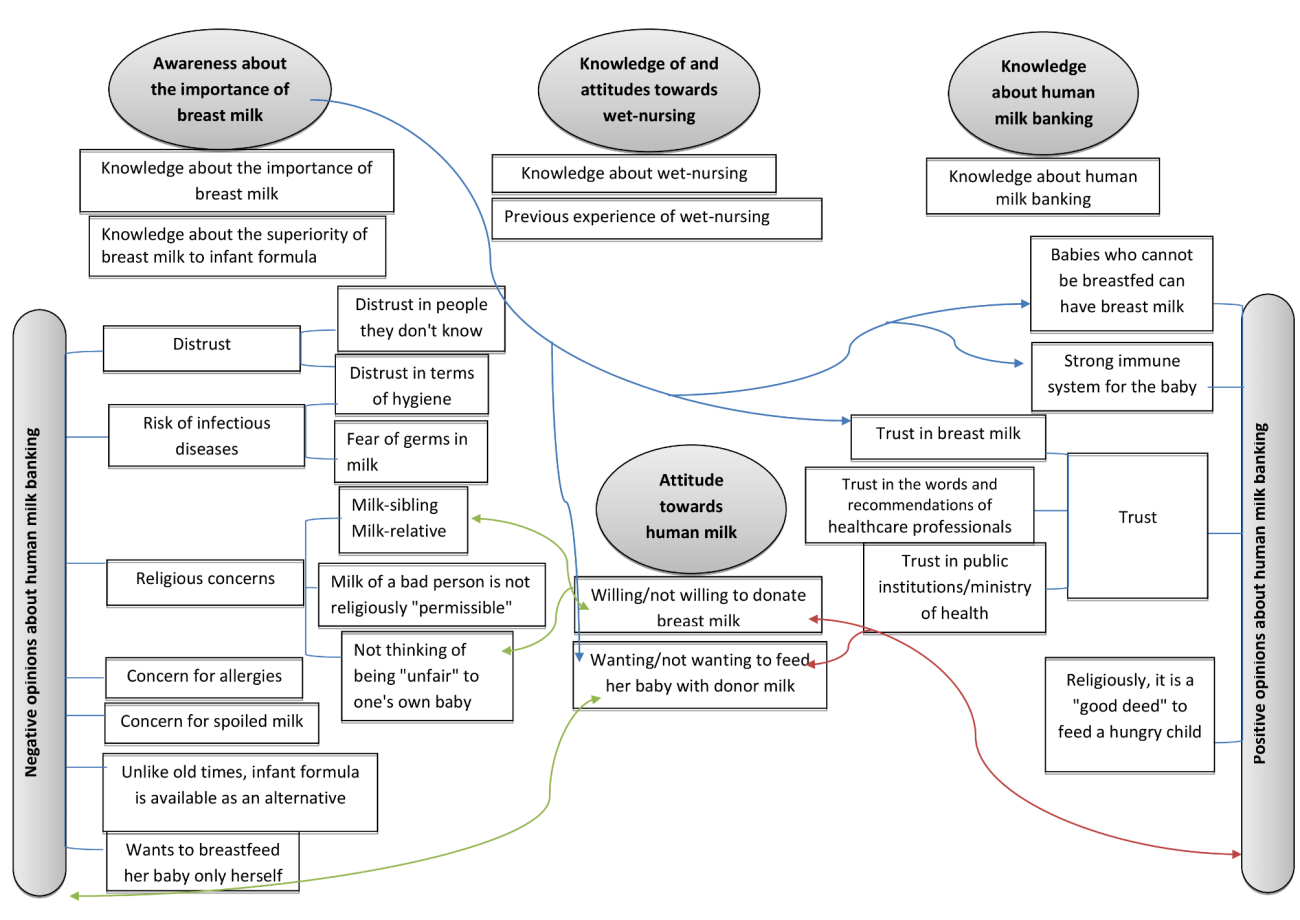
Conceptual framework for qualitative data

Analysis of the qualitative data was performed manually using thematic content analysis. The data obtained from the IDIs were first transcribed. Another researcher checked 20% of the transcripts. After all interview transcripts were read separately by two researchers (AM, CVA), common and different concepts were identified, a code was determined for each concept, and codes were grouped under thematic titles. Discrepancies were addressed by discussing differences in coding during research meetings and amending coding practice until coding was consistent. Then a conceptual framework was created for the themes (Fig. 1). For the selection of quotes from respondents, statements that were given frequently or statements that were unique, as well as statements that were similar or not similar were taken into consideration.

Quantitative data were evaluated using the SPSS 24.0 statistics program. For descriptive analysis, numeric variables are shown as mean and standard deviation, and categorical variables are shown as numbers and percentages. Bivariate analysis was conducted to examine the association between dependent and independent variables, and crude odds ratios (COR) and their 95% confidence intervals (CI) were calculated. Then, all variables that had *P*-values less than 0.05 in the bivariate analysis were included in the multivariate binary logistic regression model to identify explanatory variables that were associated with the attitudes of women towards HMB. Multivariate binary logistic regression was performed using the enter approach. Ninety-five per cent CIs were used and significance was set at *p* < 0.05 in the analyses.

## Results

Based on the qualitative study, a conceptual framework with six main themes including awareness of the importance of breast milk, knowledge and attitudes toward wet nursing, knowledge about HMB, attitudes toward HMB, positive opinions about HMB, and negative opinions about HMB was created.

The sociodemographic characteristics of the women (n = 271) who participated in the survey are shown in Table 1. The mean age of the women included in the quantitativestudy was 26.82 ± 4.89 years; 84.5% lived in nuclear families and 44.6% had one child (Table 1).

**Table 1:** Sociodemographic characteristics of survey participants (n = 271)

		n	%
Age	19– 29	200	73.8
	30– 49	71	26.2
Education status	Primary or secondary school	128	47.2
	College	95	35.1
	University	48	17.7
Working status	Housewife	175	64.6
	Employed	96	35.4
Family type	Nuclear family	229	84.5
	Extended family	42	15.5
Refugee	Yes	13	4.8
	No	258	95.2
Number of children	1	121	44.6
	2	120	44.3
	≥ 3	30	12.2

Fifteen women (n = 33) included in the qualitative study were refugees. These women were aged 21 to 40 years and had one to three children.

The women’s knowledge and attitudes toward breastfeeding, wet nursing, and HMB are shown in Table 2.

**Table 2:** Women’s knowledge of and attitudes towards breastfeeding, wet nursing and human milk banking (n = 271)

		n	%
How did you feed your youngest baby in the first six months?	Only breast milk	182	67.2
	Both	82	30.3
	Only infant formula	7	2.6
Have you ever heard about wet nurses?	Yes	240	88.6
	No	31	11.4
Have you ever breastfed other people’s babies?	Yes	34	12.5
	No	237	87.5
Would you willing to be a wet nurse for another baby?	Yes	211	77.9
	No	60	22.1
Has your baby had a wet nurse?	Yes	21	7.7
	No	250	92.3
If you don’t have enough milk, will you feed your baby with somebody else’s milk?	Yes	93	34.3
	No	177	65.3
Have you ever heard about human milk banking?	Yes	68	25.1
	No	203	74.9
Would you think about donating your breast milk that your baby does not need to a human milk bank?	Yes	157	57.9
	No	69	25.5
Would you get milk from a human milk bank if you did not have enough milk or if you could not breastfeed?	Yes	75	27.7
	No	196	72.3
Would you want a human milk bank founded in Turkey?	Yes	144	53.1
	No	39	14.4
	Undecided	88	32.5

### Awareness of women about the importance of breast milk

Sixty-seven point two per cent of the women reported that they exclusively breastfed their babies in the first six months. Only 2.7% of the mothers reported that they did not breastfeed their babies in the first six months.

Almost all of the native and refugee women reported that they were aware of the importance and benefits of breastfeeding, and the superiority of breast milk to infant formulas. *“There is nothing like breast milk; better than everything, it’s so good. Breast milk is very important for the baby’s development. Instead of giving formula, breast milk is much more precious. I think breast milk can be in no way harmful. Breast milk, how do they say, like antibiotics for the health, I think it can no way be harmful.* [D.D., 23 years, N] *A refugee mother said: “Every baby who needs it should get breast milk. It is a good thing. Breast milk is healthier, I exclusively breastfed my baby, no formula, only breast milk.”* [S.B., 38 years, R].

### Women’s knowledge of and attitudes towards wetnursing

Eighty-eight point six per cent of the women had heard about wet nursing, and 12.5% had an experience of breastfeeding someone else’s baby. Seventy-seven point nine per cent reported that they could be wet nurses for other babies, 7.7% reported that their babies had had wet nurses, and 34.3% reported that other people could breastfeed their babies if they did not have enough milk.

In IDIs, most of the mothers said that they had known of wet nursing as a concept for a long time although they had no first-hand experience with wet nursing. *“Back in the old days, mothers used to breastfeed each other’s babies when they did not have enough breast milk. Like milk siblings, I used to hear stories about these from my mother.“* [E.K., 22years, N].

Some of them also said that they have had personal experience with wet nursing. *“For example, my neighbor breastfed my child in the beginning. My mother-in-law breastfed this neighbor’s children and my child was breastfed by them, so it’s all a twist of faith.“* [N.K., 22 years, N] Similarly, refugee women reported that they knew about wet nursing and had experienced wet nursing in their lives. *“My mother-in-law used to talk about this when we were living in the village in Syria; then I breastfed my big sister’s daughter. Old people, our elderly, used to talk about; there used to be a lot of wet nurses.“* [Z.A., 23 years, R].

### Women’s knowledge about human milk banking

Seventy-four point nine per cent of the women reported that they had never heard of HMB. Among those who reported that they had heard about HMB, 65.7% heard about it on social media, 20.9% heard about it from healthcare professionals and 13.4% heard about it from their friends.

Although some of the women reported that they had heard about HMB during IDIs, no native or refugee women had any specific knowledge about how HMBs work. *“I have never heard about human milk banks; I have heard about eye banks, blood banks, but never heard about this.”* [L.D., 32 years, N].

### Women’s attitudes towards human milk banking

Fifty-seven point nine per cent of the women were willing to donate breast milk, whereas only 27.7% were willing to use donor milk for their babies. Fifty-nine per cent of the women reported a positive attitude towards HMB (donor or recipient), and 53.1% of the respondents reported that they would prefer to have an HMB in Turkey; 32.5% were indecisive about this.

In IDIs, women generally stated that they would donate their milk to an HMB but would not take milk from the HMB for their children. *“I would donate breast milk but I would not give donor milk to my child. I would think about how they collect and store such milk. I think formula would be healthier. I am a little obsessed about hygiene, so I would not be comfortable.“* [K.Ş., 33 years, N] *“I think this is a good idea; babies who could not be breastfed could have breast milk instead of formula. I think this would be more logical, but I don’t know if I would take milk from the bank.“* [N.Y., 22 years, R].

### Women’s negative opinions about human milk banking

Fifty-nine per cent of the women (160 women) who participated in the survey reported any type of negative opinion. Of the women who reported negative opinions, 76.3% gave religious concerns; 52.5% feared infectious diseases; 42.5% reported that they did not trust people; 22.5% reported that there were infant formulas today as alternatives to breast milk; 17.5% reported fear of allergies; 6.9% reported that they wanted their babies to be breastfed only by them; and 5.6% reported fear of spoiled milk as their reasons for their negative opinions about HMB.

In IDIs, women’s negative opinions about HMB were mostly about “receiving donor milk”. During IDIs, no one reported a negative attitude about “donating milk” except for religious concerns. “*Because there is a possibility of becoming milk siblings, I neither donate nor receive milk. I would not donate; I mean if there had been no such thing as milk siblings, I would have both donated and received milk. Let’s say they will get married in the future, how will I know, so it’s a sin. So, instead of giving milk of somebody who I have never met, I’d rather feed my child with milk of somebody I know. Of course, I would not want to deprive that child of milk.”* [H.D., 33 years, N].

In particular, native women spoke of the fear of infectious diseases as the reason for their negative attitude towards receiving donor milk from an HMB. There were no refugee women who gave this as the reason. *“No, I would not feed my baby with donor milk. I would not feel comfortable, I don’t know how I can explain. I would not want to give somebody else’s milk; I would worry that my baby would get infected, sick. For me it is better to give formula; I mean, it does not make sense to me.“* [N.E., 34, N].

Another negative opinion was the feeling of “distrust”. During IDIs, women expressed distrust in two different ways: “distrust in people they do not know” and “distrust because of infectious diseases”. Native women often underlined the above-mentioned statements, whereas most of the refugee women gave “distrust in people they do not know” as the reason for their negative attitudes. *“I don’t know whether I would trust and receive milk? I guess I would not want to give donor milk to my children. I mean, let’s face it, we don’t know what kind of people donate milk. I don’t know what’s in it, of course, we know that breast milk is natural but can we trust anyone with our child? No. Giving somebody else’s milk is the same thing. I think we can’t do that. Of course, I would want to give the milk of people whom I know and trust. I would feed my child with their milk and I would give my milk to their children.* [A.T., 28 years, N]

### Women’s positive attitudes towards human milk banking

Forty-one per cent of the women (111 women) who participated in the survey reported any type of positive opinion. Seventy-two per cent of the women with positive attitudes toward HMB gave the reason that babies who could not be breastfed could still have breast milk; 65.8% gave the reason that they trusted breast milk; 58.6% gave religious reasons; and 40.5% reported that they trusted healthcare professionals / the ministry of health.

IDIs showed that there were women with a positive attitude towards HMB. Positive attitudes were mostly based on knowing the “importance of breast milk”. One of the main reasons for positive attitudes among native and refugee women toward HMB was that “babies who cannot be breastfed can still benefit from breast milk.“ *“I wish we could have places like this (*HMB*); thank God my children never needed wet nurses. I always breastfed myself; I didn’t even give formula milk but there are many children who cannot be breastfed. Let those who do not have breast milk have breast milk”.* [B.A., 27 years, N] *“Breast milk is very important, those who have give to those who don’t. This is especially good for abandoned children. I feel very sad for them, I wish they could have breast milk at least until they are 6 months old”.* [D.A., 28 years, R]

Additionally, although religious factors were generally the reasons for negative attitudes, they also played a role in positive attitudes. The IDIs found that both native and refugee women thought that it was a “good deed” in a religious sense to feed a baby in need. *“I don’t see anything bad in this. If you ask why, for example, if a 1-month-old baby cannot be fed with breast milk, it is a very bad sin. That child needs breast milk. It is better to give the child breast milk. Let’s say, God bless, think about a baby who cannot be breastfed and suffer in a hospital. My baby can be breastfed, comfortable, but that baby there is hungry, this is a sin.“* [A.T., 28 years, N] *“It is a very good deed to feed a hungry child, if that child stays hungry, then it is a sin. God forbid; if I give milk, that child can maybe live. If there was a bank here, I would donate.“* [B.S., 23 years, R].

In addition to these factors, trust in healthcare professionals and public health institutions was given as the reason for positive attitudes in native women. *“If it is approved by the ministry of health, by you, then it is good for me. But if it is something done by a normal citizen, I will not feel comfortable. I mean, I think they always check and quality control everything, packaging and then allow.* [G.B. 29 years, N]

### Factors for attitudes of women towards human milk banking

Factors for attitudes of women toward HMB are shown in Table 3. The odds of having positive attitudes toward HMB were 4.19 times higher in homemaker women (95% CI 2.0, 8.76) compared with those who worked; 4.77 times higher in women with three or more children (95% CI 1.25, 8.15) compared with those who had fewer children; 6.12 times higher in women who had a positive attitude towards wet nursing (95% CI 3.14, 9.63) than those who did not; and 2.68 times higher in those who had previously heard about HMB (95% CI 1.24, 5.79).

**Table 3:** Factors associated with having a positive attitude about human milk banking

		n(%)	Crude OR (95% Cl)	Adjusted OR (95% Cl)
**Age**	19– 29	108 (54.0)	1	
	30– 49	52 (73.2)	**1.71 (1.12, 2.59)**	1.22 (0.58, 2.56)
**Education status**	Primary or Secondary	86 (67.2)	**1.47 (1.08, 1.98)**	0.80 (0.42, 1.89)
	College	55 (57.9)	1.07 (0.64, 1.78)	
	University	19 (39.6)	1	
**Working status**	Housewife	121 (69.1)	**3.27 (1.95, 5.50)**	**4.19 (2.0, 8.76)**
	Employed	39 (40.6)	1	
**Family type**	Nuclear family	135 (59.0)	1	
	Extended family	25 (59.5)	1.01 (0.68, 1.51)	
**Number of children**	1	63 (52.1)	1	
	2	71 (59.2)	0.99 (0.60, 1.61)	
	≥ 3	26 (86.7)	**1.68 (1.03, 2.74)**	**4.77 (1.25, 8.15)**
**How did you feed your youngest baby in the first six months?**	Only breast milk	108 (59.3)	1.95 (0.42, 8.91)	
	Both	49 (59.3)		
	Only infant formula	3 (42.9)	1	
**Ever heard about wet nursing?**	Yes	156 (66.1)	**5.78 (2.28, 14.6)**	2.83 (0.59, 3.62)
	No	4 (11.4)	1	
**Positive attitude towards wet nursing**	Yes	152 (71.0)	**5.06 (2.64, 9.67)**	**6.12 (3.14, 9.63)**
	No	8 (14.0)	1	
**Ever heard about HMB?**	Yes	46 (71.9)	**2.08 (1.13, 3.83)**	**2.68 (1.24, 5.79)**
	No	114 (55.1)	1	

## Discussion

This study intended to determine the attitudes of native Turkish and refugee women living in a rural area toward HMB. Almost all of the women included in this study agreed on the importance of breast milk and the majority reported that they breastfed their babies. Studies conducted in Turkey also underlined that awareness about the importance of breast milk among women was high [14, 15, 19]. Breast milk and breastfeeding are supported in Turkey and the Ministry of Health has been running the Baby-Friendly Hospital Initiative of the United Nations Children’s Fund (UNICEF), and breastfeeding is encouraged by law and supported by all public organizations and non-governmental organizations [20].

In this study, the majority of the women had heard about wet nursing and reported that they would be willing to be wet nurses; only one in eight, however, had experienced this. Although the use of wet nurses has decreased significantly today [7], mothers’ willingness to be wet nurses is also supported by the literature [19, 21].

More than two-thirds of the women included in this study reported that they had never heard of HMB before. Similar studies report that women have higher awareness in countries where HMBs are used [21–23] and levels of awareness about HMB are low in other countries [24–26]. Although the respondents’ awareness level of HMB was low, at the end of the study, more than half of the respondents supported the founding of an HMB in Turkey. Not having an HMB system in Turkey could be the reason why their level of knowledge was low.

Although women are more willing to donate breast milk, they are quite hesitant about feeding their babies with milk from the bank. Only one out of four women reported that they would give donor milk from the bank to their babies. Previous studies conducted in hospitals or online in Turkey found similar results [14, 15]. In the present study, the reasons behind the negative attitudes of women toward HMB were religious concerns, fear of infectious diseases, and distrust of people they did not know. Fear of infectious diseases was specifically mentioned by native Turkish women in IDIs, and religious concerns and distrust were reported both by native Turkish and refugee women. The native women and refugees were all Muslims, which explains why they had similar religious concerns. Studies conducted in other Muslim countries also reported religious concerns as the main reason for negative attitudes [6, 26]. Similar to our study findings, studies conductedin similar cultures reported that women had the cultural belief of distrusting people they did not know and that personality traits of the donor mother could be transferred to the child through donor milk, in addition to religious concerns [26, 27]. A study conducted in Turkey found that the main reasons for unwillingness to accept donor milk were fear of infectious diseases, quality and hygiene concerns, and personal inconvenience [28]. In studies conducted in Africa, HIV infection risk was a major concern, and women worried that even diseases that were not infectious could infect their babies and poison them [29, 30]. In a study conducted in Austria, women reported that the process of donating and receiving milk could be time-consuming, tiring, and painful [22]. The reason why there are such differences between countries could be differences in socio-cultural and economic structures, health outcomes, and health priorities. This could mean that when planning a milk banking system, adapting it according to religious and cultural values and considering the safety of human milk in terms of infectious diseases could increase its acceptability by the public.

Different from other studies [24, 26], in the present study, religious factors were the reason behind both negative attitudes and positive attitudes. More than half of the women with positive attitudes reported that giving breast milk to a baby was important for religious reasons. The reason for this difference could be the high level of awareness and knowledge of the women in the region about the importance of breast milk. Therefore, we can conclude that explaining the importance of breast milk in detail to women will increase the acceptability of HMB.

The present study showed that there were factors that could be used to increase positive attitudes toward HMB. Positive attitudes toward HMB were higher in women who reported positive attitudes toward wet nursing and in those who reported that they had previously heard about HMB. Several studies support these findings [24, 31, 32]. In conclusion, as women’s knowledge about donating and receiving breast milk increases, their positive attitudes toward HMB also increases. Other factors that showed a significant relationship with HMB in our study were employment status and the number of children. Some studies reported that there was a relationship between positive attitudes and age, occupation and education levels, having a premature baby, and breastfeeding [14, 33, 34]. Different from other studies, our study found that homemaker women and women with a higher number of children had positive attitudes that could be caused by their long experience and more time spent on breastfeeding and child care.

As in the rest of the world, HMB would play a vital role also in our country making it possible for babies who cannot be breastfed for any reason to have access to invaluable breast milk. Even in countries such as Brazil where there are successfully established HMB systems, a widespread effort is still needed to encourage women to donate breast milk [35]. Methods that can provide solutions and relieve religious and cultural concerns and issues about the safety of milk should be suggested.

### Limitations of the study

As an inherent characteristic of cross-sectional studies, the quantitative part is limited to explaining causality. Because there were insufficient refugees to achieve a good comparison with native women in the area, comparisons could only be made between refugees and native women in the qualitative part of the study. Additionally, because the study was conducted in a rural area, the study results cannot be generalized to the entire region. In our study, there was only information on people who were registered in primary healthcare institutions. However, even if they are not officially registered, the records and contact information of people who present to the institution only once are kept, so it can be thought that the results of the study represent the women in the region. The study has some major strengths, however. The mixed-methods used in the study allowed us to investigate different aspects of the study subject. Including refugee women in the qualitative part will be guiding because this is the first study on this subject that includes refugees. Being population-based is another strength of the study. The majority of the related studies in the literature evaluated women who had just given birth in hospitals, thus our study is important also in this sense.

## Conclusion

According to the findings of this study, the respondents’ attitudes toward HMB are affected by religious beliefs, cultural beliefs, and concerns about the safety of breast milk collected and stored via HMB. Refugees and native Turkish women were found to have similar religious concerns. On the other hand, the importance and benefits of breast milk were acknowledged by both groups. These findings should be taken into consideration in HMB initiatives and in activities to increase acceptance by the public in Turkey and countries with similar socio-cultural characteristics.

## Data Availability

The datasets used and / or analysed during the current study are available from the corresponding author on reasonable request.

## Abbreviations

HMB: Human Milk Banking
IDI: Depth interviews
UNICEF: United Nations Children’s Fund
WHO: World Health Organization
